# A Peculiar Mutation Spectrum Emerging from Young Peruvian Patients with Hepatocellular Carcinoma

**DOI:** 10.1371/journal.pone.0114912

**Published:** 2014-12-11

**Authors:** Agnès Marchio, Stéphane Bertani, Teresa Rojas Rojas, Franco Doimi, Benoît Terris, Eric Deharo, Anne Dejean, Eloy Ruiz, Pascal Pineau

**Affiliations:** 1 Institut Pasteur, Unité Organisation Nucléaire et Oncogenèse, Paris, France; 2 INSERM, U993, Paris, France; 3 Université de Toulouse, UPS, UMR152 PHARMADEV, Université Toulouse 3, Toulouse, France; 4 Institut de Recherche pour le Développement, UMR152 PHARMADEV, Lima, Peru; 5 Aix-Marseille Université, UMR912 SESSTIM INSERM-IRD-AMU, Centre d′Epidémiologie et de Santé Publique des Armées, Marseille, France; 6 Instituto Nacional de Enfermedades Neoplásicas, Departamento de Patología, Banco de Tejidos Tumorales, Lima, Peru; 7 Assistance Publique-Hôpitaux de Paris, Hôpital Cochin, Service d'Anatomie et Cytologie Pathologiques, Paris, France; 8 Institut de Recherche pour le Développement, UMR152 PHARMADEV, Vientiane, Laos; 9 Instituto Nacional de Enfermedades Neoplásicas, Departamento de Cirugía en Abdomen, Lima, Peru; CRCL-INSERM, France

## Abstract

Hepatocellular carcinoma usually afflicts individuals in their later years following longstanding liver disease. In Peru, hepatocellular carcinoma exists in a unique clinical presentation, which affects patients around age 25 with a normal, healthy liver. In order to deepen our understanding of the molecular processes ongoing in Peruvian liver tumors, mutation spectrum analysis was carried out on hepatocellular carcinomas from 80 Peruvian patients. Sequencing analysis focused on nine genes typically altered during liver carcinogenesis, i.e. *ARID2*, *AXIN1*, *BRAF*, *CTNNB1*, *NFE2L2*, *H/K/N-RAS*, and *TP53*. We also assessed the transcription level of factors involved in the control of the alpha-fetoprotein expression and the *Hippo* signaling pathway that controls contact inhibition in metazoans. The mutation spectrum of Peruvian patients was unique with a major class of alterations represented by Insertions/Deletions. There were no changes at hepatocellular carcinoma-associated mutation hotspots in more than half of the specimens analyzed. Furthermore, our findings support the theory of a consistent collapse in the *Hippo* axis, as well as an expression of the stemness factor NANOG in high alpha-fetoprotein-expressing hepatocellular carcinomas. These results confirm the specificity of Peruvian hepatocellular carcinoma at the molecular genetic level. The present study emphasizes the necessity to widen cancer research to include historically neglected patients from South America, and more broadly the Global South, where cancer genetics and tumor presentation are divergent from canonical neoplasms.

## Introduction

Hepatocellular carcinoma (HCC), the major form of primary liver cancer, is a leading cause of death from malignancy, ranking at the third position worldwide [1]. HCC remains a deadly disease, generally diagnosed at an advanced stage, when surgical intervention is no longer possible because of tumor extension. HCC incidence is known to vary widely throughout the world depending on region with areas of high incidence, such as Eastern Asia and sub-Saharan Africa, and areas of low incidence, like Northern Europe and North America [2]. High incidence areas of HCC correspond grossly to zones with distribution of two major risk factors, i.e. chronic infection with hepatitis B virus (HBV) and aflatoxin B1 (AFB1) intoxication [3]. Elsewhere, chronic infection with hepatitis C virus (HCV), excessive alcohol use, or dysmetabolic conditions dominate HCC epidemiology, neighboring sometime with additional endemic risk factors, such as hemochromatosis in Western Europe or alpha-1 antitrypsin deficiency in Scandinavia. Meanwhile, large geographic areas, such as Eastern Europe, Northern and Central Asia, Latin America, and the Caribbean, have not been fairly scrutinized regarding prevalent risk factors and common liver cancer clinical presentation. Likewise, molecular epidemiology of HCC in these areas remains largely unknown. The issues raised here represent a serious matter for global public health.

As with most types of cancer, the incidence of HCC has heightened dramatically in recent past, and the HCC epidemic will continue to grow exponentially for coming decades according to recent estimates by the World Health Organization [1], [2]. The burden of HCC increases first and foremost in the Global South, with nearly 85% of HCC cases and 64% of HCC-related deaths monitored worldwide occurring in developing countries. Therefore, action is required by both the scientific community and public health decision-makers to address this plight. Like most of the Global South, Latin America is facing this burgeoning cancer issue [4].

Peru is the South American country with the highest rate of primary liver cancer [1]. Surgeons of the National Cancer Institute of Peru (INEN), the major Peruvian cancer center, have recently described a locally frequent, but elsewhere unusual, form of HCC affecting children, adolescents, and young adults [5]. We then reported that age-specific distribution of HCC in Peruvian patient population was delineating a bimodal distribution, with a first peak of incidence at age 25 and a second one at age 64 [6]. These HCC cases appearing in younger patient population were characterized by a tremendous elevation of alpha-fetoprotein (AFP) tumor marker serum concentration, the absence of cirrhosis in 95% of the cases, and the presence of an associated risk factor [mostly HBV surface antigen (HBsAg) carriage] in merely 50% of cases [6].

It is generally admitted that the biology of cancers in adolescents and young adults differs dramatically in terms of pathways alteration from those striking later in lifespan [7]. Moreover, due to their rarity, our knowledge about early-life HCCs is lacking. In order to gain insights into the molecular changes occurring in the unusual clinical presentation of HCC in young Peruvian individuals, we conducted a comparative analysis of mutations with age of 80 HCCs obtained from patients admitted at INEN, i.e. 41 persons below age 40 (<40) and 39 persons above or equal age 40 (≥40). Thirty-one exons covering 17 kb in nine genes considered as mutation hotspots in HCC were analyzed. The purpose of the present study was to determine whether genetic variations exist between younger and older Peruvian HCC patients and to compare this spectrum with data published from elsewhere. In addition, expression of transcriptional regulators of the *AFP* gene and of some crucial members of the *Hippo* signaling pathway was monitored. We present therein the first molecular analysis of HCC from an indigenous population of the Western Hemisphere. Interestingly, our data suggest that these tumors are significantly divergent from the patterns already described in Eastern Asia, sub-Saharan Africa, or Western Europe.

## Materials And Methods

### 1. Ethics statement

Prior to their inclusion, all patients received information regarding the purpose and conduct of the study. Each patient provided specific written consent for the storage of his or her information and samples in the Tumor Bank of INEN, and their use for research. The study conforms to the ethical principles contained in the Declaration of Helsinki, and was approved by the Human Subjects Committee of INEN (Protocol Number #INEN10-05).

### 2. Patient recruitment and clinical specimen collection

A series of 80 patients with HCC attending INEN between August 2006 and March 2011 was enrolled in the study. HCC patients were treated in the Department of Abdominal Surgery of INEN by anatomic liver resection, i.e. systematic removal of the tumoral liver segments confined by portal branches with surgical margin [5]. 50 mg of both HCC and non-tumor liver (NTL) matched pair tissues (HCC/NTL) were promptly harvested from the resected surgical piece, flash frozen with liquid nitrogen, then kept at minus 80°C for long-term storage. Surgical report recorded tumor presentation and additional pathophysiological comments. Follow-up medical consultations and phone interviews were used to assess patient condition following intervention.

### 3. Liver cancer classification

Cancer type and staging, as well as Edmonson-Steiner tumor grade (E.S.), were assessed on hematoxylin–eosin-stained liver biopsy sections according to the recommendations of the American Joint Committee on Cancer [8]. Anatomic pathology diagnosis of HCC was confirmed twice independently by trained liver pathologists both in Lima and Paris.

### 4. Mutation detection

Tissues were pounded under liquid nitrogen and then digested at 37°C for 8 hours in tissue lysis buffer containing proteinase K and sodium dodecyl sulfate (SDS). Genomic DNA was extracted twice with phenol and once with chloroform, precipitated in ethanol, and resuspended in TE buffer (10 mM Tris; 0.1 mM EDTA; pH 8.0). Mutations and polymorphisms of tumor DNA were detected by direct PCR amplification and subsequent sequencing performed using the BigDye Terminator procedure (Applied Biosystems), as described [9]. Mutations were confirmed by sequencing the second DNA strand. For every case of a tumor DNA alteration, the germline DNA from parent NTL was subsequently analyzed using the same procedure, in order to assess the somatic nature of the mutant. Only those alterations that were present in HCC DNA and absent from NTL DNA were scored as *bona fide* HCC-associated mutations. Point mutations were searched in 31 exons covering 17 kb in nine genes, i.e. *tumor protein p53* (*TP53*), *catenin (cadherin-associated protein) beta 1* (*CTNNB1*), *axin 1* (*AXIN1*), *v-raf murine sarcoma viral oncogene homolog B* (*BRAF*), *AT rich interactive domain 2* (*ARID2*), *nuclear factor erythroid 2-like 2* (*NFE2L2*), and *Harvey* (*H-*), *Kristen* (*K-*), and *neuroblastoma* (*N-*) *rat sarcoma viral oncogene homolog* (*RAS*) genes. The DNA primer pairs used in PCR assays are detailed in S1 Table.

### 5. Quantitative PCR assays

Real-time reverse-transcriptase PCR (qRT-PCR) assays were performed essentially as described [10] (see S1 Text for details). Relative quantitation calculations were done according to the ΔΔC_T_ method using the geometric mean of three housekeeping genes as references: *tripartite motif containing 44* (*TRIM44*), *hydroxymethylbilane synthase* (*HMBS*), and *lipase maturation factor 2* (*LMF2*) complementary DNA transcripts. The three reference genes were selected among 12 constant genes arising from a previous array analysis of 70 HCC and nine NTL samples to which were applied algorithms. The DNA primer pairs used in qRT-PCR assays are described in S2 Table.

### 6. Western blot analysis

Western blot analysis was performed on eight HCC/NTLs, using primary antibody to Yes-associated protein 1 (YAP), phosphorylated (p-) YAP (both CST), and glyceraldehyde 3-phosphate dehydrogenase (GAPDH) (Abcam) (see S1 Text for details). Relative protein expression levels were established by normalization on GAPDH protein expression.

### 7. Hepatitis viruses detection

Infections with HBV and HCV were assessed from serum of HCC patients with electro-generated chemiluminescence Elecsys HBsAg II and Anti-HCV II assays (Roche Diagnostics), using antibodies against HBsAg and HCV antibody (anti-HCV), respectively. HBV DNA was detected in tissues by TaqMan assay (Pa03453405_s1) (Life Technologies). HCV and hepatitis delta virus (HDV) RNAs were detected in tissues by nested reverse transcriptase PCR assays (see S2 Table for details).

### 8. Serum AFP level detection

AFP was monitored for its preoperative serum concentration in all patients during the week before surgery, using electro-chemiluminescence immunoassay (ECLIA) Elecsys AFP kit (Roche Diagnostics), according to the manufacturer's instructions. Diagnostic AFP threshold level for HCC detection at first hand was set at 400 ng/mL.

### 9. Statistical analyses

The age distribution of the patients was analyzed using a Gaussian mixture model for detecting bimodality and calculating bimodality index (BI) [11], [12]. Pearson's second skewness coefficients were calculated as described [13]. Comparisons between prevalence of alterations were performed using the Chi-square and Fisher's exact tests. Continuous variables were analyzed with Student's *t*-test and Mann-Whitney U test. Statistical analyses were performed with an alpha significance level 0.05, using InStat package (GraphPad Software Inc.). Class discovery was performed by unsupervised hierarchical clustering, using the DNA-Chip Analyzer (dChip) software version 2010 (HSPH). Mean values are presented with standard deviation (±SD).

## Results

### 1. Patient clinical demography and HCC-associated risk factors

**Figure 1:**
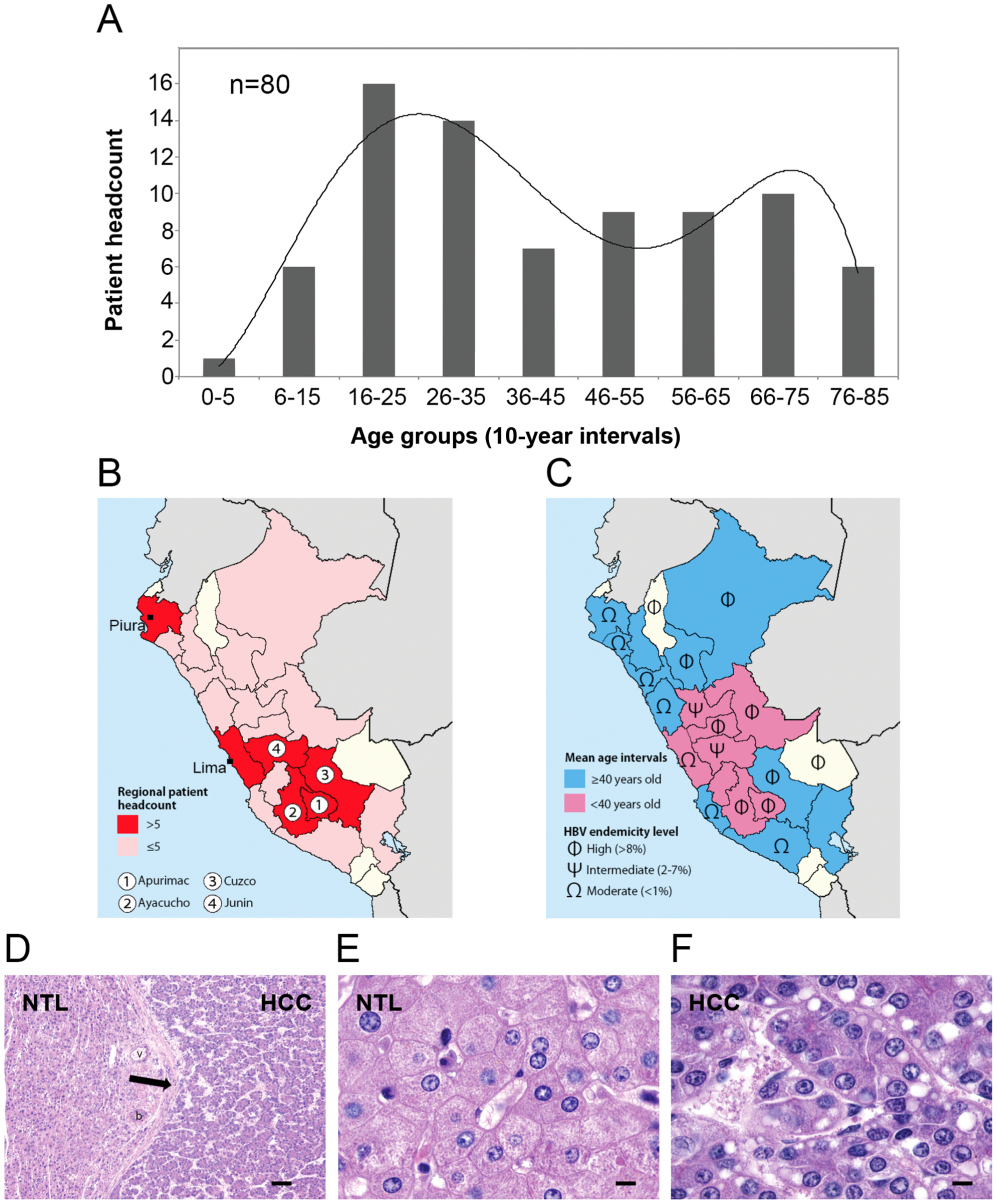
Occurrence of HCC in Peru presents an age-based clinico-epidemiological polarity. (**A**) Histogram presenting the distribution of the present patient population according to patients' age at the time of diagnosis. X-axis displays age (10-year bin); y-axis displays the headcount of patients for a given age group (n = 80). Straight line represents the histogram curve fitting with a Gaussian mixture function (BI = 1.8). This bimodal distribution concurs with the observation previously made in Peru [6]. (**B,C**) Distribution maps of the population size (B) and mean age (C) of the patients according to their regional origin. (**B**) Regions for which patient headcount was ≤5 and>5 are choropleth mapped in pink and red, respectively. The southern-central Andean area encompassing the regions of Apurimac (#1), Ayacucho (#2), Cuzco (#3), and Junin (#4) is the area from where was originating the larger subset of <40 patients. (**C**) Regions for which mean age was <40 and ≥40 years old are choropleth mapped in magenta and blue, respectively. Φ indicates highly endemic regions of HBV infection (>8%); Ψ indicates regions of intermediate endemicity (2-7%); Ω indicates regions of moderate endemicity (<1%), as described [14]. (**B,C**) Regions for which headcount was null are choropleth mapped in off-white. (**D–F**) Hematoxylin–eosin-stained liver sections from a 37-year-old Peruvian female individual with an 18-cm-diameter multinodular, moderately differentiated HCC. (**D**) Both NTL (left) and HCC (right) tissues presented under low magnification (40x). The arrow indicates the fibrotic tissues of the capsule enclosing HCC nodules. b: bile duct; v: vein. Scale bar; 100 µm. (**E**) Presentation of the normal NTL tissue under high magnification (100x). (**F**) Presentation of the E.S.2 HCC tissue with a trabecular pattern under high magnification (100x). (**E,F**) Scale bars; 10 µm.

**Figure 2:**
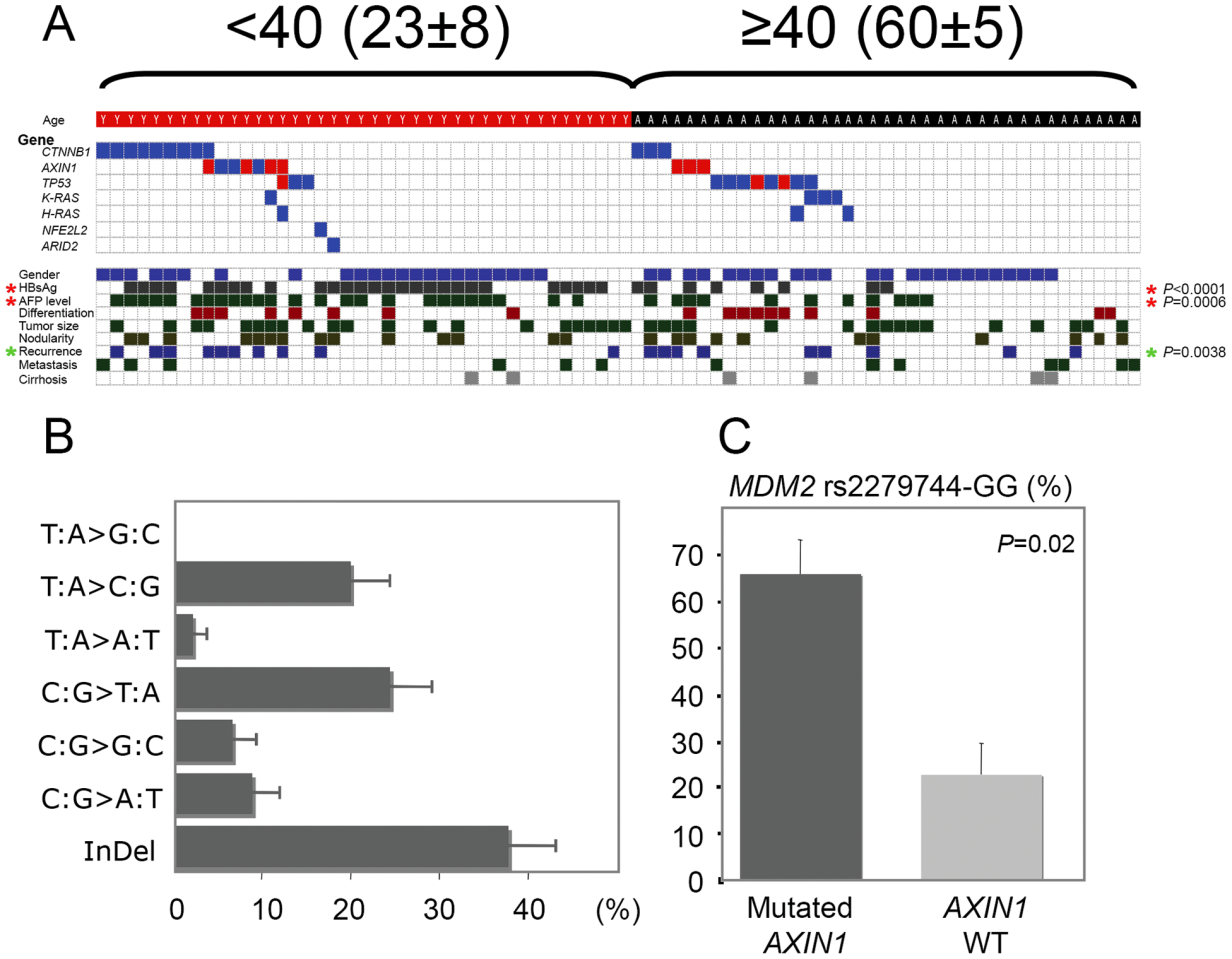
The mutation spectra emerging from Peruvian HCCs display age-based peculiar genetic features. (**A**) Mutations found in HCCs from <40 (left) and ≥40 (right) Peruvian patients, as see in *CTNNB1*, *AXIN1*, *TP53*, *K-RAS*, *H-RAS*, *NFE2L2*, and *ARID2* genes (n = 80). Mutation presence is indicated on the upper panel by both blue and red check marks. Red check marks correspond to inactivating mutations, i.e. nonsense or frame shift mutations. Corresponding clinico-pathological features are mentioned on the lower panel. Check marks correspond to (from top to bottom): male sex; HBsAg(+); serum AFP level above the median; poorly differentiated tumor; ≥17 cm-diameter tumor; multinodular tumor; recurrence within 12 months following anatomic liver resection; metastatic cancer; and cirrhotic NTL. Red asterisks indicate significant differences between <40 and ≥40 patients both for HBsAg(+) (*P*<0.0001) and serum AFP level (*P* = 0.0006). Green asterisk indicates significant difference between HCCs with mutation(s) and HCCs with no mutation (*P* = 0.038). (**B**) Mutation spectrum of Peruvian HCC (n = 80). X-axis displays percentage of genetic alteration; y-axis displays each of the six classes of base substitution and Insertions/Deletions (InDels). (**C**) Bar chart illustrating the association between *AXIN1* gene mutation and *MDM2* GG genotype at the rs2279744 allele. (**B,C**) Error bars represent the standard errors of the counts.

The overall *sex-ratio* (Male:Female) was 1.4, with 58.7% of men and 41.3% of women (Table 1). The mean and median ages of the overall cohort of patients were respectively 42±20 and 39 years old, ranking from age 1 to age 83 (Fig. 1A and Table 1). The age distribution of the patients displayed bimodality with two frequency modes (BI = 1.8) (Fig. 1A). The skewness coefficients of age distribution were rather low with 0.4 and minus 0.6 for the <40 and ≥40 patient groups, respectively, indicating that both age distributions were centered around their respective mean (Table 1). Patients originated from 20 of 25 Peruvian regions, with major subsets of individuals originating from the northern and central coastal areas (i.e., Lima and Piura regions) (n = 17; 21%) and in the southern-central Andes (i.e., Apurimac, Ayacucho, Cusco, and Junin regions) (n = 29; 36%) (Fig. 1B). This latter subset was significantly overrepresented in regard to the total population of Peru, in which the aggregated population of these four regions represents only 11.6% (*P* = 0.0003). Geographic disparities in mean age for diagnosis and development of HCC were observed with younger mean ages recorded evenly in the central regions of Peru (Fig. 1C). NTL tissues were scored as normal in 56% of the cases (n = 45) (Fig. 1D–F). A necro-inflammation indicating hepatitis was present in 28% of the patients (n = 23), and full-fledged cirrhosis was found in only 7.5% of cases (n = 6). Other conditions, such as hepatic fibrosis or steatosis, sometimes present concomitantly or with chronic hepatitis, were found in 11.2% (n = 9) and 12.5% (n = 10) of cases, respectively. Persistent infection with HBV characterized by seropositivity for HBsAg [HBsAg(+)] was the principal risk factor, present in 42% of the patients (n = 34) (Fig. 2A). HBsAg(+) HCC patients were significantly younger than HBV non-carrier ones (30±16 vs. 50±20 in mean age; *P* = 0.0008) (Fig. 2A). This situation is usually observed from the onset of HBsAg(+) medical history in high-incidence areas of HCC [15], [16]. Molecular detection of HBV DNA by PCR revealed a high rate of occult infection in Peruvian patient population, as viral DNA was found in 74% of the patients (n = 59). Consistently, the proportion of <40 patients (n = 37) with detectable HBV DNA in liver tissue was significantly higher than the corresponding subset of ≥40 patients (n = 22), i.e. 90% vs. 56%, respectively (*P* = 0.01). Serology for HCV was positive in only 2.5% of the cases of the overall cohort (n = 2), i.e. one in <40 patients and one in ≥40 patients. Presence of HCV RNA was confirmed by PCR in liver tissue for both cases. A single patient (1.2%) was positive by PCR for HDV. No history of alcohol abuse was determined. Serum AFP concentrations were above diagnostic threshold in 65% of the cases (n = 52), and in a twilight zone above normal values in another subgroup of nine patients (11%). HBsAg(+)-associated HCCs tended to be significantly associated with higher circulating AFP values (*P* = 0.0485), and a significantly larger subset of <40 patients was characterized by high serum AFP levels compared with ≥40 patients, i.e. 84% vs. 47%, respectively (*P* = 0.0006) (Fig. 2A). The median size of the tumors was 13 cm in diameter, and a vast majority of them (75%) was well or moderately differentiated (E.S.1 and E.S.2, respectively). In 40% of cases, tumors were multinodular at time of surgical resection (n = 32). We observed no correlation between tumor size and grade and serum AFP values. Patients born in southern-central Andes tend to form a distinct clinico-biological entity when compared with individuals originating in the coastal regions (Fig. 1B,C). Compared with the coastal patient population, Andean patients were younger (*P* = 0.0353), and were more often carriers of HBV markers both at the serological (64% vs. 29%; *P* = 0.0028) and molecular (97% vs. 69%; *P* = 0.0036) levels. They also displayed lower tumor grade (e.g., E.S.1 and E.S.2), as none of them presenting poorly differentiated tumor (*P* = 0.0003).

**Table 1:** Demographical and clinico-pathological features of the HCC patient population analyzed.

		Overall	<40 subset	≥40 subset
**Cohort**	Headcount	80 (100%)	41 (51%)	39 (49%)
**Age**	Mean±SD	42±20	23±8	60±5
	Median	39	22	61
	Range	[1–83]	[1]–[39]	[40–83]
**Gender**	Female	33 (41%)	18 (44%)	15 (38.5%)
	Male	47 (59%)	23 (56%)	24 (61.5%)
	*Sex-ratio* (M:F)	1.4	1.3	1.6
**E.S.**	1,2	62 (77%)	33 (80.5%)	29 (74%)
	3	18 (23%)	8 (19.5%)	10 (26%)
**Cirrhosis**	Yes	6 (7.5%)	2 (5%)	4 (10%)
	No	74 (92.5%)	39 (95%)	35 (90%)
**HBsAg(+)**	Yes	35 (43%)	27 (66%)	8 (20.5%)
	No	45 (57%)	14 (34%)	31 (79.5%)
**HBV DNA**	Yes	59 (74%)	36 (88%)	23 (59%)
	No	21 (26%)	5 (12%)	16 (41%)
**Anti-HCV**	Yes	2 (2.5%)	1 (2.5%)	1 (2.5%)
	No	78 (97.5%)	40 (97.5%)	38 (97.5%)
**Tumor size (cm)**	Mean±SD	14±6	15±6	14±6
	Median	13	14	13
**Multinodularity**	Yes	25 (31%)	14 (33%)	11 (28%)
	No	55 (69%)	27 (67%)	28 (72%)
**Metastasis**	Yes	13 (16%)	6 (14.5%)	7 (18%)
	No	67 (84%)	35 (85.5%)	32 (82%)
**Recurrence**	Yes	19 (24%)	10 (25.5%)	9 (23%)
	No	61 (76%)	31 (74.5%)	30 (77%)
**Liver enzymes (IU/L)**	ALP (mean±SD)	279±240	280±262	279±222
	AST (mean±SD)	109±125.5	129±151	86±85.5
	GGT (mean±SD)	62.5±71.5	76.5±87	47±45.5
**AFP (ng/mL)**	Median	13,700	33,450	239
	IQR	113,181	337,777	17,567

Abbreviations: AFP = alpha-fetoprotein; ALP = alkaline phosphatase; AST = Aspartate transaminase; E.S. = Edmonson-Steiner tumor grade; GGT = Gamma-glutamyl transpeptidase; HBV = hepatitis B virus; HBsAg = HBV surface antigen; HCC = hepatocellular carcinoma; HCV = hepatitis C virus; IQR = interquartile range; M:F = ratio of men to women; ±SD = standard deviation of the mean. <40 and ≥40 subsets are defined as patients below age 40 and those equal or above age 40, respectively. Multinodularity indicates HCC with more than one intrahepatic nodule. Recurrence indicates patients who developed a new HCC within the following 12 months after anatomic liver resection. Percentages are expressed as proportion of the total patient population for the considered parameter.

### 2. Mutation analysis

We analyzed common liver cancer-associated mutation hotspots in the present series of 80 HCCs (see S3 Table for details). A total of 31 exons spanning 17 kb of genomic DNA within nine genes were screened on each samples. *BRAF* and *N-RAS* genes did not present any genetic alterations in all specimens analyzed (n = 80). Thirteen *TP53* mutations were found in 14% of the cases (n = 11) (Fig. 2A). Alterations were present in five *TP53* gene exons with exon 6 affected in half of the cases. Only one AFB1-induced *TP53* R249S mutation was detected in a poorly differentiated (E.S.3) HCC from a 49-year-old male patient originating from the northern region of Piura (1.2%) (Fig. 1B). Interestingly, this patient (PER37) had been submitted to an apparently intense mutagenic stress as three different point mutations were found in his *TP53* gene (see S3 Table for details). A proline at the polymorphic codon 72 in exon 4 of *TP53* gene was present in 61% of the cases (n = 49), and 15% of the HCC specimens were homozygous for this codon (n = 12). Such allelic distribution, with a predominant proline, is a characteristic feature of populations living around the equator [17]. *CTNNB1* and *AXIN1 Wnt* axis member genes, both common mutation targets in HCC, were also investigated. Mutation rate of the *CTNNB1* gene was rather low with merely 15% (n = 12), compared with mutation rates observed in European HCC patients (Fig. 2A) [18]. Interestingly, 58% of these mutations were deletions (n = 7). *AXIN1* gene was investigated for presence of mutation in seven exons covering 77% of the *AXIN1* open reading frame, i.e. 1,988 on 2,589 full-length nucleic acid sequence. 12.5% of the HCC specimens were found to carry mutation (n = 10). Overall, the *Wnt* axis was altered in 26% of the Peruvian HCC cases (n = 21). Four prominent members of *Ras* signaling pathway (i.e., *H/K/N-RAS* and *BRAF* genes) were submitted to mutation screening. 9% of the HCC specimens displayed mutations in *Ras* axis, four affecting *K-RAS* and three on *H-RAS* (n = 7). Remarkably, all four *K-RAS*-mutated HCCs were unusual I21M mutants [19]. Two additional genes, i.e. *NFE2L2* and *ARID2*, recently proposed as mutational targets in HCC were investigated [20]. Only one somatic mutant was found for each gene (1.2%). The nucleotide mutation spectrum of the 45 somatic mutants identified displayed a peculiar pattern (Fig. 2B). Surprisingly, the main class of mutation was InDels, a type of alteration usually prevalent at low level in HCC patients originating from Europe or the Far East. Mutations were found in 45% of the patients (n = 36), leaving the remaining 44 cases without any molecular clue regarding the ongoing molecular process. *MDM2* rs2279744-GG genotype is correlated with a reduced activity of tumor suppressor p53 in the corresponding tumors [21], [22]. *AXIN1-*mutated and *MDM2* rs2279744-GG genotypes were significantly associated in HCC patients (Fig. 2C). 72% of *AXIN1*-mutated HCC samples were GG homozygotes at SNP309, whereas this proportion was only 34% in HCC specimens lacking *AXIN1* mutation (*P* = 0.0218).

### 3. Correlation between genetic alterations and clinico-pathological features

**Figure 3:**
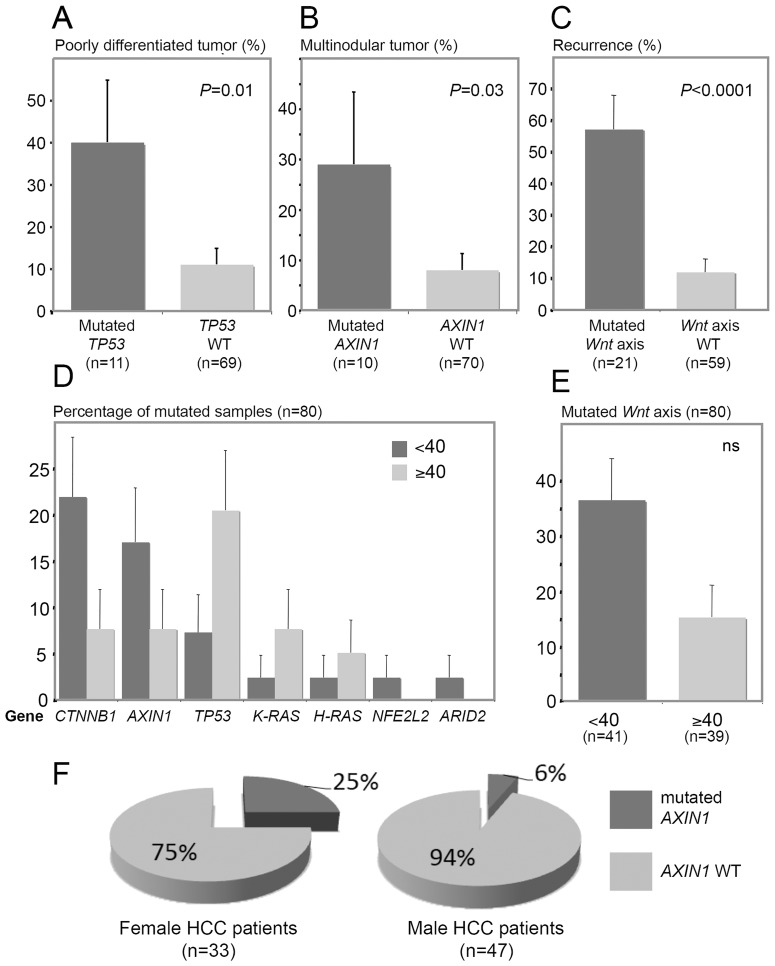
Age-based tumor phenotype and clinical pattern of the Peruvian HCCs are correlated to specific somatic mutation rates. (**A–E**) Bar charts illustrating the association between HCC-related gene mutation rates (%) and tumor clinical presentation. (**A–C,E**) Black bars represent the mutation rate; grey bars represent the wild-type allele (WT) rate. (**A**) WT and mutation rates of *TP53* gene in E.S.3 HCCs (*P* = 0.01). (**B**) WT and mutation rates of *AXIN1* gene in multinodular HCCs (*P* = 0.03). (**C**) WT and mutation rates of *Wnt* axis in recurring HCCs (*P*<0.0001). (**D**) Bar chart of the mutation rates of *ARID2*, *AXIN1*, *CTNNB1*, *H-RAS*, *K-RAS*, *NFE2L2*, and *TP53* genes in <40 and ≥40 patients (black and grey bars, respectively). (**E**) Bar chart presenting mutation rate of *Wnt* axis in <40 and ≥40 HCCs. Black and grey bars represent <40 and ≥40 patient rates, respectively. (**A–E**) Error bars represent the standard errors of the counts. (**F**) Pie charts for both mutation and WT rates of *AXIN1* gene in HCCs of female (left chart) and male (right chart) patients. Black sectors represent the *AXIN1* mutation rates; grey sectors represent the rates of *AXIN1* WT.

To shape an intelligible landscape of the HCC affecting Peruvian patients, we subsequently tried to correlate virology and genetic features with the clinical presentation of the disease. As expected, *TP53* mutations were statistically associated with a poor tumor differentiation (*P* = 0.0045) (Figs. 2A and 3A). In addition, multinodular liver tumors were preferentially associated with specific genetic traits, e.g. *AXIN1* mutation (*P* = 0.0309) and *MDM2* GG polymorphism at SNP309 (60.8% vs. 33.3%; *P* = 0.0417) (Figs. 2A and 3B). Both *CTNNB1* (58% vs. 16%) and *AXIN1* (54% vs. 16%) mutations were significantly enriched in patients undergoing a subsequent recurrence of their tumor (*P* = 0.0045 and *P* = 0.0022, respectively) (Fig. 2A). *Wnt* axis was altered in 72% of patients undergoing a tumor recurrence, while this signaling pathway was mutated in merely 15% of the patients with a relapse-free survival (*P*<0.0001) (Figs. 2A and 3C). We also detected differences in mutation rates of seven genes according to patients' age (Fig. 3D). On the same hand, *Wnt* axis was found altered in 15 tumors from <40 patients, whereas it was mutated in only six tumors from ≥40 patients (36.5% vs. 15.4%; *P* = 0.042) (Figs. 2A and 3E). Moreover, *AXIN1* mutations were significantly more frequent in liver tumors striking in women than in those of men (*P* = 0.043) (Fig. 3F). The geographic localization of the patients was not associated with any specific genetic alterations, concurring with the fact that the population structure of Peru is genetically homogenate [23]. Albeit not reaching the level of significance, a deviation to this observance was a trend for *AXIN1* mutant enrichment in patients originating from the southern-central regions of Peru compared with those coming from the coastal provinces of the country (19.3% vs. 0%; *P* = 0.0571) (Fig. 1B,C).

### 4. Involvement of the *Hippo* axis in tumor size control

**Figure 4:**
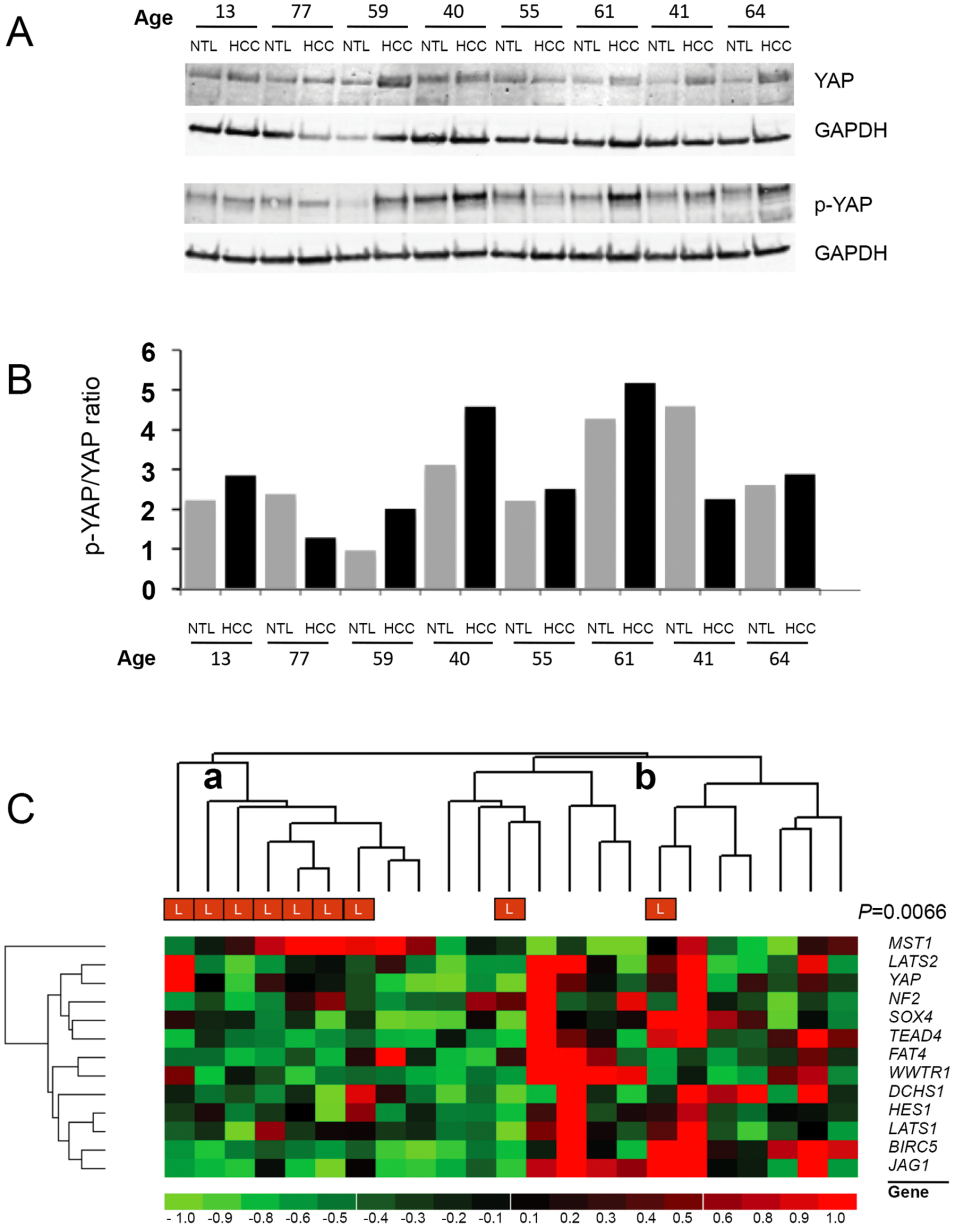
The *Hippo* axis is down-regulated during the development of massive HCC in Peruvian patient. (**A**) Western blot analysis of total cell extracts prepared from HCC/NTL of eight Peruvian patients (ranking from age 13 to age 77). Cell extracts were separated by SDS-PAGE and electrophoretically transferred onto nitrocellulose membrane. The blots were probed using antibodies to YAP (upper panel), p-YAP (lower panel), and GAPDH (both panels). GAPDH was used as a loading control. (**B**) Bar chart representation of the p-YAP/YAP ratio in these eight HCC/NTLs. Relative quantification of YAP and p-YAP protein expression was measured on western blots using ImageJ software and the blotted signals were normalized to GAPDH expression before forming the ratio of the densitometric values of bands containing YAP and p-YAP proteins. (**C**) Heat map of an unsupervised hierarchical clustering of expression of 13 *Hippo* axis genes [i.e., *baculoviral IAP repeat containing 5* (*BIRC5*), *dachsous cadherin-related 1* (*DCHS1*), *FAT atypical cadherin 4* (*FAT4*), *hes family bHLH transcription factor 1* (*HES1*), *jagged 1* (*JAG1*), *large tumor suppressor kinase 1* (*LATS1*) and *2* (*LATS2*), *macrophage stimulating 1* (*MST1*), *neurofibromin 2* (*NF2*), *SRY-box 4* (*SOX4*), *TEA domain family member 4* (*TEAD4*), *WW domain containing transcription regulator 1* (*WWTR1*), and *YAP* genes] in Peruvian HCCs (n = 23). Results are expressed as HCC/NTL mRNA expression ratios. The left-most cluster (a) is highly enriched in huge tumors (L) (*P* = 0.0066) and displays a general collapse of *Hippo* axis expression (except for *MST1* and *STK4* genes) when compared with the right-most cluster (b). L corresponds to the largest tumors (≥17 cm-diameter) present in the third tertile of the patient cohort (n = 23).

Massive tumor size was frequently observed in the present series of patients (median of 13 cm-diameter in tumor size). This uncommon feature prompted us to investigate the molecular bases of such clinical trait. *Hippo* axis is considered as a key player in the control of organ size and contact inhibition in metazoans [24]. The *Hippo* axis-associated YAP transcription factor (TF) is considered as a major effector of cell growth in HCC [25]. We thus hypothesized that the *Hippo* axis could be affected in the huge HCCs developed by Peruvian people. We decided to investigate the presence of both total YAP and p-YAP protein in a subset of HCC/NTLs associated with the development of either smaller or larger liver tumor (n = 8). Both YAP and p-YAP protein levels did not show significant difference (Fig. 4A–B). In order to identify a possible alteration within the *Hippo* axis-associated gene network, we conducted an expression survey of 13 *Hippo* pathway gene members in a subset of 23 HCC/NTLs associated with development of 7- to 26-cm-diameter HCCs. Unsupervised clustering analysis of gene transcription indicated a consistent collapse of *Hippo* axis expression in huge tumors, suggesting that this pathway is crucial for the massive tumor phenotype frequently observed in Peruvian patients (Fig. 4C). However, the general repression of *Hippo* axis appears to be a delayed event in tumor development as it occurred essentially in tumors of the last tertile for size (i.e., ≥17 cm-diameter).

### 5. Monitoring of the *AFP* gene expression in massive HCC

**Figure 5:**
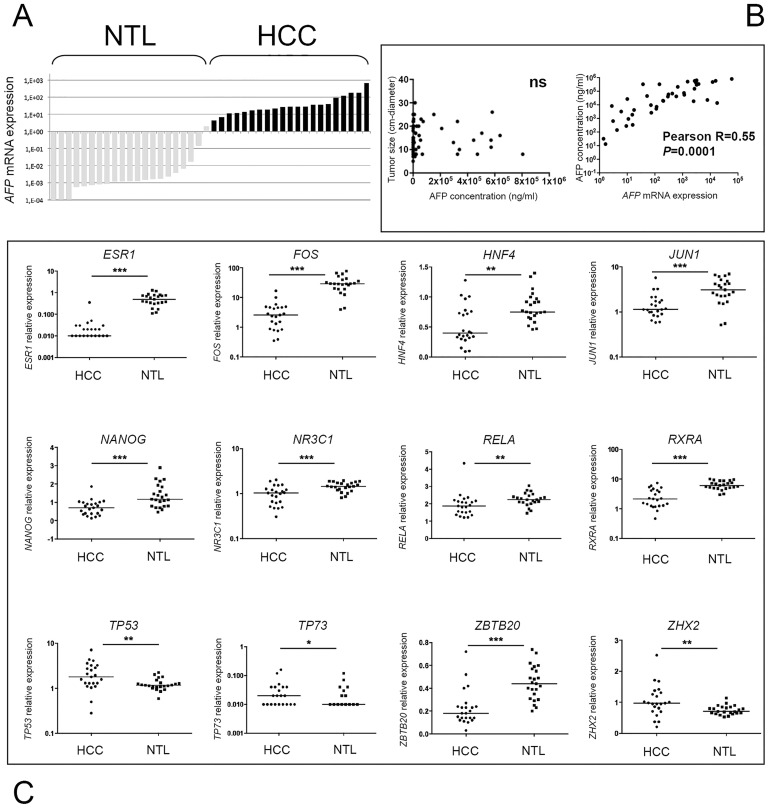
Serum AFP concentration in Peruvian HCC is concomitantly correlated to *AFP* gene overexpression and down-regulation of TF-encoding genes involved in *AFP* gene control. (**A**) Bar chart displaying the *AFP* gene transcription levels obtained by qRT-PCR from 23 matched pairs of NTL (grey bars; left) and HCC (black bars; right) tissues from Peruvian patients. (**B**) Scatter plots showing the relationship between (left) the serum AFP concentration (x-axis) and the tumor size (y-axis) (ns); (right) the *AFP* mRNA expression (x-axis) and the serum AFP concentration (y-axis, n = 40, *P* = 0.0001). (**C**) Dot plots of the relative expression of 12 TF-encoding genes (i.e., *ESR1*, *FOS*, *HNF4*, *JUN*, *NANOG*, *NR3C1*, *RELA*, *RXRA*, *ZBTB20*, *TP53*, *TP73*, and *ZHX2*) controlling *AFP* gene expression in HCC/NTL as measured by qRT-PCR (n = 23). ***P<0.0001; **P<0.001; *P<0.05.

**Figure 6:**
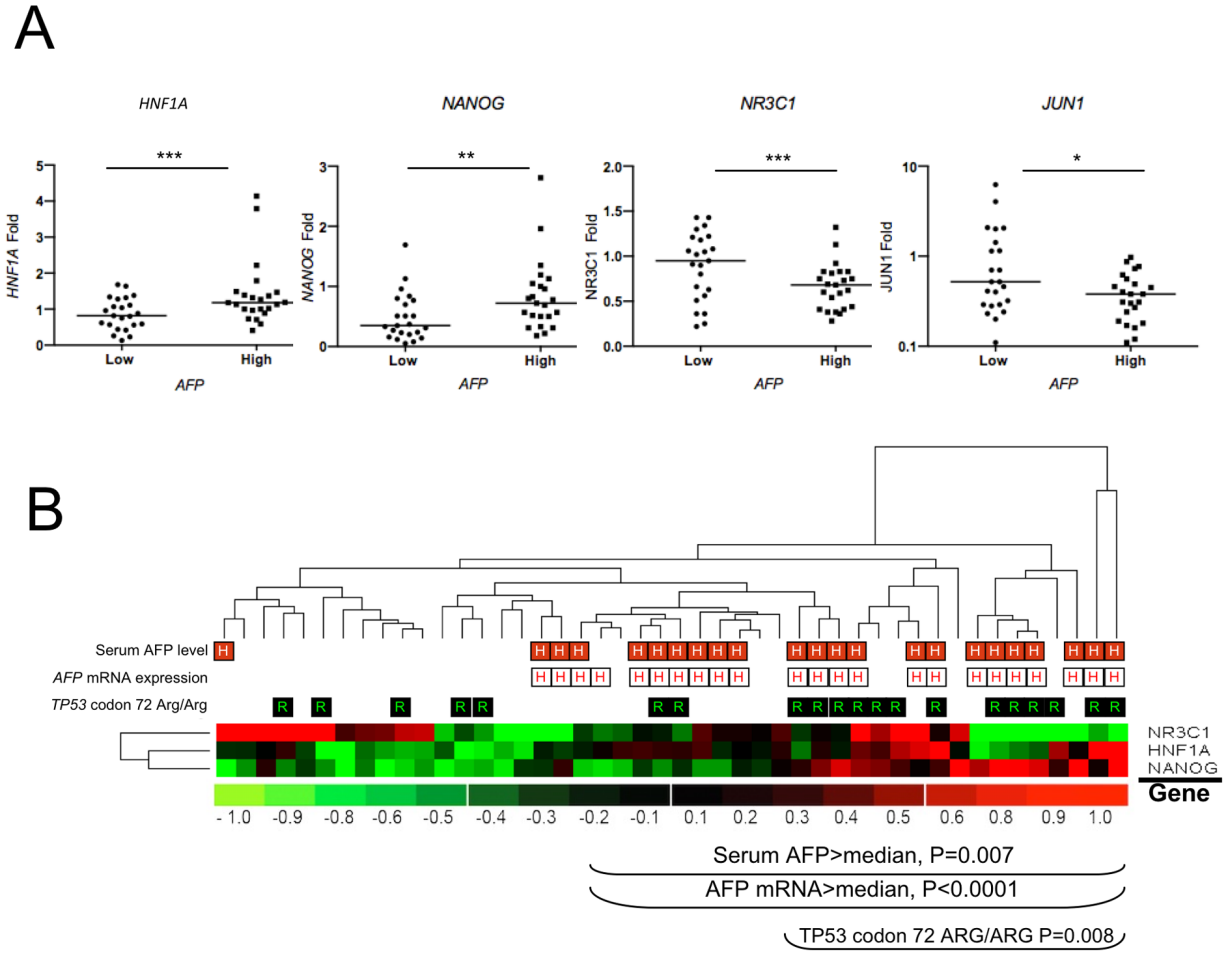
High expression of both alpha-fetoprotein transcript and polypeptide is correlated to *NANOG* gene transcription in massive Peruvian HCC. (**A**) Dot plots indicating expression folds of four TF-encoding genes controlling *AFP* gene expression in high (n = 23) and low (n = 23) *AFP*-expressing HCC tumors. Folds of expression level of *AFP* gene are defined according to their position above (high) or below (low) the median *AFP* gene expression level of the cohort. ***P = 0.01; **P = 0.02; *P = 0.07 (ns). (**B**) Heat-map of the unsupervised hierarchical clustering for *HNF1A*, *NANOG*, and *NR3C1* expression, TFs involved in *AFP* gene regulation in Peruvian HCCs (n = 46). The pervasive expression of *NANOG* gene correlates with high serum AFP concentration (red-squared H) (*P* = 0.007) and *AFP* mRNA level (white-squared H) (*P*<0.0001), whereas expression of *NR3C1-GR* gene and down-regulation of *NANOG* gene is preferentially observed in low AFP-expressing tumors. Arg/Arg homozygosity at codon 72 of *TP53* (rs1042522) (green-squared R) is associated with high serum AFP concentration and *AFP* mRNA level, as well as increased *NANOG* gene expression (*P* = 0.008).

Exceeding serum AFP concentration is another hallmark of the tumorigenic process ongoing in Peruvian patients and can provide some substantial insights regarding the molecular bases of the disease [6]. Given the young age of many patients, we first assessed whether *AFP* mRNA could be expressed in NTL of <40 patients with high value of circulating AFP. A subset of 23 HCC/NTLs was screened for their *AFP* gene expression by qRT-PCR assays. There was no significant *AFP* gene expression in NTLs, with a mean 1,700-fold ratio between HCC and parent NTL tissues (Fig. 5A). We then assessed whether tumor sizing was influencing serum AFP concentration. In keeping with seminal work in the field, no correlation was found between tumor dimension and serum AFP levels in the blood (Fig. 5B) [26]. By contrast, circulating AFP concentration was correlated with the levels of *AFP* mRNA in tumor cells (*P* = 0.0001) (Fig. 5C). Postnatal repression of *AFP* gene is primarily achieved at the transcriptional level [27], [28]. A plethora of TFs have been claimed to be involved in up- or down-regulation of the *AFP* gene promoter activity. Among them stand TFs known to be crucial in tumor development or cancer regression, such as ESR1, FOS, JUN, HIF1a, MYC, RELA, TP53, and TP73. We thus decided to explore the gene expression level of 23 TFs controlling directly or cooperatively the promoter activity of *AFP* gene in 23 HCC/NTLs (Table 2). A salient decrease in transcript abundance was observed in HCC compared with NTL for nine genes assessed, i.e. *ESR1*, *FOS*, *HNF4*, *JUN1*, *NANOG*, *NR3C1*, *RELA*, *RXRA*, and *ZBTB20* (Fig. 5C and Table 2). In parallel, a significant increase of gene expression in tumor specimens was observed for *TP53*, *TP73*, and *ZHX2* (Table 2). Finally, 11 genes were not differentially transcribed between HCC and parent NTL (Table 2). Overall, mean changes in TF mRNA expression were rather mild, exceeding two-fold only for *ESR1* (12-fold down), *FOS* (6-fold down), *RXRA* (2.2-fold down), and *TP73* (4.8-fold up). Interestingly, we noticed that a subset of eight TF genes investigated, i.e. *CTCF*, *HNF1A*, *JUN1*, *NANOG*, *NR3C1*, *ONECUT1*, *ZBTB20*, and *ZHX2*, tended to be differentially transcribed on this training samples set when stratified according to the fold of *AFP* gene expression. To validate this inference, we decided to increase the number of specimens analyzed with another 23 HCC/NTLs. Three of the TF genes scrutinized, i.e. *HNF1A*, *NANOG*, and *NR3C1*, were confirmed as significantly differentially transcribed in low and high *AFP*-expressing HCC tumors, whereas *JUN1* gene expression displayed only an *infra* significance trend (Fig. 6A). Surprisingly, the transcription of some TF genes was either repressed (e.g., *NANOG* and *JUN1*) or unchanged, such as *HNF1A* (data not shown), in HCC tissues when compared with parent NTL tissues (Fig. 5A). Up-regulation of *AFP* gene transcription was, as a consequence, associated with those samples undergoing only a mild down-regulation of the TF genes. Unsupervised hierarchical clustering of *NR3C1* (encoding for the glucocorticoid receptor, GR), *HNF1A*, and *NANOG* gene expression indicated that *NANOG* up-regulation was the most correlated with both high serum AFP concentration and *AFP* mRNA transcript abundance in Peruvian HCC patients, whereas *NR3C1* up-regulation was associated with lower serum AFP levels (Fig. 6B). Interestingly, both high serum AFP levels and *NANOG* up-regulation expression were significantly associated with the Arg/Arg genotype of *TP53* codon 72 (rs1042522) (*P* = 0.008).

**Table 2:** Relative expression in Peruvian HCC of 23 TFs controlling the *AFP* gene transcription.

Gene	Full name	Genomic location	HCC:NTL(mean±SD)	*P*-value
*CTCF*	*CCCTC-Binding Factor*	16q21-q22.3	0.95±0.3	ns
*ESR1*	*Estrogene Receptor 1*	6q25.1	0.08±0.2	0.0001
*FOS*	*FBJ Murine Osteosarcoma Viral Oncogene*	14q24.3	0.17±0.2	0.0001
*FOXA1*	*Forkhead Box A1*	14q12-q13	1.04±0.8	ns
*FOXA2*	*Forkhead Box A2*	20p11	1.32±0.9	ns
*HIF-1A*	*Hypoxia Inducible Factor 1, Alpha Subunit*	14q24.2	1.31±0.9	ns
*HNF1A*	*HNF1 Homeobox A*	12q24.2	0.92±0.4	ns
*HNF4*	*Hepatocyte Nuclear Factor 4*	20q13.12	0.68±0.4	0.001
*JUN*	*Jun Proto-Oncogene*	1p32-p31	0.81±1.2	0.0001
*MYC*	*Avian Myelocytomatosis Viral Oncogene Homolog*	8q24.21	1.69±1.6	ns
*NANOG*	*Nanog Homeobox*	12p13.31	0.67±0.5	0.0001
*NR3C1-GR*	*Nuclear Receptor Subfamily 3, Group C, Member 1*	5q21.3	0.71±0.2	0.0001
*ONECUT1*	*One Cut Homeobox 1*	15q21.3	1.71±1.6	ns
*RELA*	*V-Rel Avian Reticuloendotheliosis Viral Oncogene Homolog A*	11q13	0.87±0.3	0.002
*RXRA*	*Retinoid X Receptor, Alpha*	9q13.1	0.45±0.2	0.0001
*SP1*	*Sp1 Transcription Factor*	12q13.1	1.15±0.4	ns
*SRF*	*Serum Response Factor*	6p21.1	1.24±0.7	ns
*TCF4*	*Transcription Factor 4*	18q21.1	1.20±0.5	ns
*THRA*	*Thyroid Hormone Receptor, Alpha*	17q11.2	1.69±1.1	ns
*TP53*	*Tumor Protein p53*	17p13.1	1.89±1.4	0.003
*TP73*	*Tumor Protein p73*	1p36.3	4.85±6.3	0.05
*ZBTB20*	*Zinc Finger and BTB Domain Containing 20*	3q13.3	0.57±0.4	0.0001
*ZHX2*	*Zinc Fingers and Homeoboxes 2*	8q24.13	14.1±0.6	0.004

Abbreviations: HCC = hepatocellular carcinoma; HCC:NTL = ratio of relative transcript expressions in HCC to parent non-tumor liver; ns = non significant; TF = transcription factor; ±SD = standard deviation of the mean. TF relative expressions were measured by qRT-PCR assays on HCC and non-tumor liver matched pair specimens from 23 Peruvian patients. *P*-values were obtained by exact simple logistic regression analysis.

## Discussion

HCC is a malignant tumor resulting from exposition to a plethora of different risk factors acting frequently in concomitance, but not evenly distributed globally [29]. As a consequence, HCC displays a remarkable heterogeneity in terms of patient demographical features, clinical presentation, rates of underlying cirrhosis, tumor pathology, or molecular epidemiology [30]. Thus, our understanding of the pathophysiology of the disease relies greatly on our knowledge of the ethno-geographical context. According to most recent reviews, the usual presentation of HCC in South-America is overall similar to that observed in Europe or North America with patients developing the liver tumor in the seventh decade of life, on a cirrhotic liver infected most commonly with HCV [31]. However, this pattern does not prevail in the Peruvian population and it has been reported since the 1990s that cases of early HCC are commonly encountered in Peru [5], [32], [33]. We published previously that age of HCC patients in Peru follows a bimodal distribution with a major peak of occurrence in the third decade of life [6]. This age-specific bimodal distribution was retrieved in the present series as well (Fig. 1A). A similar clinical presentation was originally reported in Mozambique, where HBV and AFB1 used to represent heavy disease burdens for the population [34]. A significant subset of the patients investigated in the present study was originating from hyper-endemic isolates of persistent hepatitis B located in central Andes regions, such as the Apurimac and Ayacucho regions (Fig. 1B) [14]. In contrast with other places as Mozambique or the Qidong region in China, it is highly improbable that AFB1 plays a significant role in Peruvian HCC [34], [35]. For instance, we found only one case of the known AFB1-induced R249S *TP53* gene mutation. Moreover, the male patient presenting this alteration was relatively old, i.e. 49 years old, with regard to the present series and was coming from the northern coastal city of Piura, distant from the central Andes area from which most of the younger patients originate (Fig. 1B).

Taken together, the bimodal distribution for age and the low *sex-ratio* represent usual hallmarks of HCC appeared on non-cirrhotic livers [6], [36], [37]. Whereas such pathophysiological pattern elsewhere represents a smaller fraction of HCC cases observed, it is the dominant phenotype in Peru, suggesting either the presence of an unusual environmental risk factor or a peculiar predisposition of the Peruvian population to liver tumorigenesis in a context of exceptional resistance to hepatic fibrogenesis.

Among the most significant traits observed in the present patient series was the association between age and hepatitis B seropositivity (Fig. 2A). HBsAg(+) was present in a majority of the cases arisen before age 40, whereas it was present in only a minority of cases above this age. Although HBsAg(+) was not associated to any specific genetic alterations in our study, it seems that HBV is acting like a booster of the liver tumorigenesis in Peruvian patients. Interestingly, liver tumor development in <40 patients who are often HBsAg(+) was frequently associated with mutations in the *Wnt* axis mutations. Such genotypic alterations are unusual elsewhere, as *CTNNB1* mutants were found preferentially in non-HBV-associated HCC [38]–[40]. However, they are strongly reminiscent of the G1 subgroups of young African women with occult HBV infection developing HCC characterized by developmental program defect and *AXIN1* mutations as described [41]. The uncommon genetic profile of Peruvian HCC was further confirmed by the conspicuous excess of deletions present in the mutation spectrum, a pattern rarely observed in sporadic cancer and never described so far in HCC (Fig. 2B) [42]–[44]. These mutations were significantly affecting more the *Wnt* axis than other signaling pathways, suggesting that the mechanism of *Wnt* axis alteration could be different from that affecting other targets in Peruvian HCC. The causes of such phenomenon have yet to be elucidated, but could be linked to environmentally induced defective activity of the DNA polymerases. For instance, carcinogenic compounds found to produce liver tumors such as N-acetyl-2-aminofluorene are known to induce such mutations [45], [46]. However, the natural occurrence of similar substance and their impact in human pathology has not yet been observed.

Although not considered as a gold standard marker, AFP remains worldwide the most useful diagnostic biomarker of HCC in routine clinical practice [47]. AFP is transiently produced during the establishment of the endoderm in embryogenesis, but its expression is reactivated during liver tumorigenesis [48]. AFP is suspected to play an active role in liver tumorigenesis either by immune modulation or its anti-estrogenic property [49]. Interestingly, both Asian and European authors have reported that high AFP level is predictive of poor survival in HCC patients [50]. AFP expression is known to vary with ethnicity in pregnancy, and was shown to reach lower levels in Hispanics than other women living in USA [51], [52]. Besides, AFP expression is modulated in acute hepatitis and by environmental conditions in human and in various animal models exposed to carcinogens [53]–[59]. The fact that exceedingly high serum AFP concentrations was often monitored in <40 Peruvian HCC patients prompted us to use AFP protein synthesis as a proxy to explore the biological process controlling liver tumorigenesis in the Peruvian population. As AFP production is primarily controlled at the transcription level, we decided to analyze the expression of the major TFs regulating *AFP* gene transcription in liver cells (Fig. 5C) [27], [28]. A majority of the genes assessed, those that encode for *AFP* transcription repressors, such as FOS, HNF4, JUN1, and ZBTB20, were down-regulated in HCC tissues, revealing a general alteration of the gene network that normally suppresses *AFP* expression in adult liver cells [60]–[62].

Among TFs that control *AFP* gene transcription in a cooperative fashion stands NANOG, a stemness factor expressed in pluripotent and cancer stem cells, and which is one of the major protein protagonists involved in the cell allostatic decision between self-renewal and differentiation [63], [64]. NANOG expression has been associated with cancer cell invasiveness and metastatic tumor presentation [65]. Intriguingly, that was not the case in the present Peruvian HCC series, in which higher *NANOG* gene expression was correlated neither to metastatic nor hepatic multinodular tumors. Notwithstanding, HCC tissues from Peruvian patients displaying higher *NANOG* gene expression were associated with heightened expression of both alpha-fetoprotein transcript and polypeptide (Fig. 6A,B). The apparently paradoxical relative decrease of *NANOG* transcript abundance in tumor tissues compared with NTL ones maybe be attributed either to a large excess of NANOG biosynthesis by proliferative endothelial cells in normal hepatic tissue, or to an aberrant state of differentiation of the tumor cells that retains a decayed stem cell phenotype together with the expression of endodermal lineage markers, such as AFP [66], [67]. Therefore, these findings supports the hypothesis that the HCC developed by the Peruvian patients, and notably by the youngest ones, can be linked to a peculiar phenomenon of hepatocyte mis-speciation or de-differentiation, plausibly involving liver cancer stem cells, as it has been previously postulated [67]–[69].

Further studies are warranted to identify the cofactors and the cellular and molecular principles that, together with HBV infection, dramatically accelerate HCC outbreak in Peruvian individuals. Various hypotheses can be formulated that take into account the specific environmental conditions prevailing in Peru, where liver flukes, poly-metal contamination, as well as unidentified toxins, may trigger hepatic carcinogenesis [70]–[72].

## Supporting Information

S1 Table **Primers used to detect point mutations in HCC from Peruvian patients.**
(XLS)

S2 Table **Assays and primers used for qRT-PCR analyses and viruses detection.**
(XLS)

S3 Table **Summary of the mutations detected in HCC from Peruvian patients.**
(XLS)

S1 Text **Supporting experimental protocols.**
(DOC)
