# Supplementary material for: A Peculiar Mutation Spectrum Emerging from Young Peruvian Patients with Hepatocellular Carcinoma
Source: PLoS One. 2014 Dec 11;9(12):e114912. doi: 10.1371/journal.pone.0114912 (PMC4263719; doi:10.1371/journal.pone.0114912)
Supplement: S1 Text — Supporting experimental protocols. (DOC) [file pone.0114912.s004.doc]

**SUPPORTING EXPERIMENTAL PROTOCOLS**

**Quantitative PCR assays**

Total RNA pools were isolated from frozen HCC and NTL tissues, using TRI Reagent® (Euromedex) and Lysin Matrix D homogenization system (MP Biomedicals), according to the manufacturer’s instructions. RNA integrity and quantity were assessed using the RNA 6000 Nano LabChip® kit (Agilent Technologies) and a 2100 Bioanalyzer (Agilent Technologies). For complementary DNA (cDNA) synthesis, 1 mg of total RNA was reverse transcribed using High-Capacity cDNA Archive kit (Applied Biosystems), according to the manufacturer's instructions. QRT-PCR assays were performed in a CFX96 qPCR machine (Bio-Rad). 10 μl qRT-PCR mixture consisting of 20 ng of cDNA product, 1X TaqMan® Universal PCR Master Mix and 1 μl of pre-validated TaqMan® Gene Expression Assay (Applied Biosystems) were incubated in a 96-well optical plate at 95°C for 10 min and amplified for 40 cycles of 95°C for 15 s and 60°C for 1 min. The threshold cycle (C_T_) data was determinate using default threshold settings. The C_T_ is defined as the fractional cycle number at which the fluorescence passes the fixed threshold. For data analysis, gene expression values were determined using the calculation of the relative quantitation of target genes normalized to a calibrator corresponding to five normal livers. QRT-PCR assays were performed in triplicate for each sample.

**Western blot analysis**

Tissues were homogenized at 4**°**C in 10 volumes (w/v) extraction buffer [50 mM Tris-HCl pH 8.0; 0.1 mM EDTA; 200 mM NaCl; 1% NP40; 10% glycerol; 200 mM NaCl; 20 mM N-ethyl-maleimide; 1x Protease inhibitor EDTA free; and PhosSTOP® Phosphatase Inhibitor Cocktail tablets (Roche Diagnostics)] using a FastPrep®-24 homogenizer (MP Biomedicals). Cell debris was removed by centrifugation at 13,000 rpm for 10 min, and protein concentration was estimated using Pierce™ BCA Protein Assay kit (ThermoScientific). 20 μg of proteins per lane were resolved on the 4-12% gradient Gel precast polyacrylamide gels (Bio-Rad) and transferred onto nitrocellulose membrane using the Trans-Blot® Turbo™ Transfer System (Bio-Rad), according to manufacturer’s instructions. Both anti-YAP and anti-p-YAP polyclonal primary antibodies were used at a dilution of 1:1,000, whereas anti-GAPDH monoclonal [6C5] antibody was used at 1:4,000. Blots were probed with appropriate fluorescent secondary antibodies (Odyssey) at a dilution of 1:10,000 and visualized using the Odyssey® CLx Infrared Imaging System and software (LI-COR Biosciences). The signal of each lane was quantified by ImageJ 1.48 program (NIH).
